# The Protective Effect of Sesamol in the Selenite-induced Experimental Cataract Model

**DOI:** 10.4274/tjo.42385

**Published:** 2017-12-27

**Authors:** Burak Turgut, İrfan Ergen, Nevin İlhan

**Affiliations:** 1 Yüksek İhtisas University Faculty of Medicine, Department of Ophthalmology, Ankara, Turkey; 2 University of Health Sciences, Dışkapı Yıldırım Beyazıt Training and Research Hospital, Department of Ophthalmology, Ankara, Turkey; 3 Fırat University Faculty of Medicine, Department of Biochemistry, Elazığ, Turkey

**Keywords:** Sodium selenite, experimental cataract, sesamol

## Abstract

**Objectives:**

To investigate the potential protective effects of sesamol in an experimental cataract model.

**Materials and Methods:**

Twenty-one Spraque Dawley rat pups were randomly assigned into three groups, seven rats in each. All the rats except for those in the control group were injected subcutaneously with a single dose of sodium selenite on postpartum day 9. On days 10-14, rats in the sham group were intraperitoneally administered 50 mg/kg/day saline solution, while rats in the sesamol group were given 50 mg/kg/day sesamol by the same route. Following cataract grading, the lenses and capsules were extracted and the mean levels of reduced glutathione (GSH), malondialdehyde (MDA), total antioxidant status (TAS) and total oxidant status (TOS) in lens supernatants were biochemically analyzed.

**Results:**

The control group did not show any development of cataract. It was found that the mean cataract grade in the sesamol group was significantly lower than that of the sham group (p<0.05). The mean GSH level and TAS in the sesamol group were significantly higher than those of the sham group while the mean TOS and MDA level in the sesamol group were significantly lower than those of the sham group (p<0.05).

**Conclusion:**

Our study shows that sesamol reduces TOS and MDA level and increases TAS and GSH level in the lens and inhibits cataract formation.

## INTRODUCTION

Cataract is the loss of transparency or clouding of the lens leading to a decrease in vision.1 Although cataract is most commonly due to aging, it may also occur due to trauma, inflammation, heredity, radiation exposure, metabolic disorders, malnutrition, and complications from other ocular pathologies.1,2 Risk factors for cataract are diabetes, smoking tobacco, prolonged exposure to sunlight, and alcohol via oxidative damage in the lens, impaired glucose metabolism, radiation damage, and toxic damage. The exact pathogenic mechanism of age-related cataract is unknown. However, it has been considered that increased free oxygen radicals, reduced antioxidant enzyme, and deterioration of the electrolyte balance in the lens play an important role in the development of cataract. Wearing sunglasses and not smoking may slow the development even if they cannot completely prevent cataract.^3,4^

Currently, the only effective treatment for cataracts is surgery, including removal of the cataractous lens and implantation of an artificial intraocular lens.^1,2,3^ It is estimated that 10 million people with cataract blindness will undergo cataract surgery by 2020.^5^ The direct annual medical cost for outpatient, inpatient, and prescription drug services related to the treatment of cataract is estimated at $6.8 billion.^6^ If cataract onset can be delayed about ten years, it is believed that the number of annual cataract surgeries will decrease up to 45%.^7^

Oxidative stress is the main mechanism in the onset and progress of cataract.^8^ According to the oxidative stress theory, free radicals lead to cataract formation via the cross-linking and aggregation of lens proteins, membrane lipid peroxidation, and activation of epithelial cell apoptosis and some damaging biochemical reactions of the lens.^9^ Although some preventive compounds are present in the lens, they are not strong enough to inhibit cataract formation. Antioxidants, especially vitamins A, C, and E, have a potential protective effect against oxidative stress in the lens by reducing and scavenging free radicals. The evidence obtained from previous studies shows that high antioxidant consumption and increased serum antioxidant levels have a protective effect against cataract formation.^10^

Sesamol (3.4-methylenedioxyphenol) is a well-known and strong antioxidant compound which is extracted from the oil of Sesamum species. Sesamol has various important biological activities besides its strong antioxidant activity, such as induction of growth arrest and apoptosis in cancer and cardiovascular cells, enhancement of vascular fibrinolytic capacity, chemoprevention, and antimutagenic and antihepatotoxic activities.^11^

To the best of our knowledge, there is no previous study in the literature investigating the use of sesamol for the prevention of cataract development. Therefore, in our study we aimed to investigate the potential protective effect of sesamol against cataract development in an experimental sodium selenite cataract model.

## MATERIALS AND METHODS

### Study Design and Ethics

This study was performed in the Department of Ophthalmology, Faculty of Medicine of our university with contributions from the Department of Biochemistry. It was funded by an unrestricted grant from the same University Scientific Research Unit. Throughout the study, the rats were maintained in the experimental research center of our university. The animals were housed in special wire-bottomed cages and in standard conditions (12-hour light-dark cycle, ventilated, constant room temperature). All animals were fed only with breast milk until postpartum 21 days, at which time they were decapitated. With approval from the ethics committee of the university, the study was carried out using one eye from each animal. All procedures were performed with strict adherence to the guidelines for animal care and experimentation as prepared by the Association for Research in Vision and Ophthalmology and Guidelines for the Housing of Rats in Scientific Institutions.

### Study Groups

The rats were randomly assigned to three groups, with seven rats in each group.

1. Control group included rats in which cataract was not induced and which did not receive any treatment.

2. Sham group included rats in which induction of cataract was performed and which were treated with saline.

3. Sesamol group included rats in which induction of cataract was performed and which were treated by sesamol.

**Figure 1:**
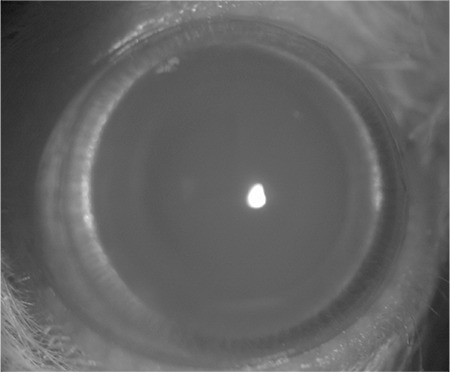
Stage 0 cataract with clear crystalline lens of one rat from control group

**Figure 2:**
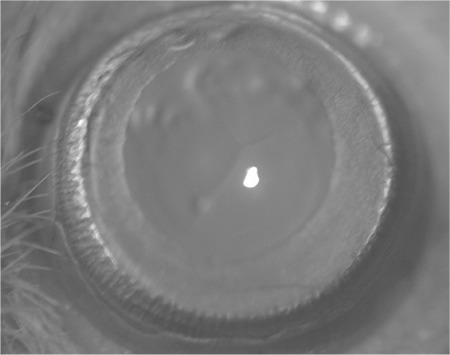
Stage 1 cataract with initial posterior subcapsular or minimal nuclear opacity

**Figure 3:**
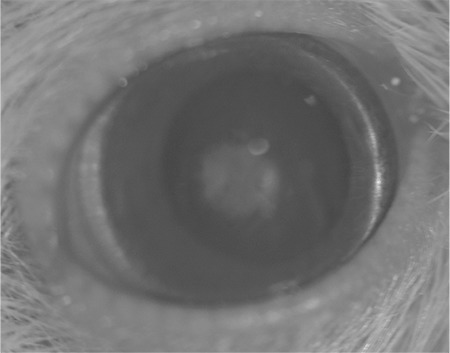
Stage 2 cataract with swollen nuclear opacity or rarely posterior subcapsular opacity

**Figure 4:**
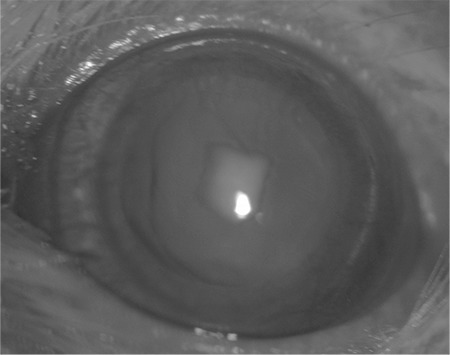
Stage 3 cataract with nuclear lens opacity

**Figure 5:**
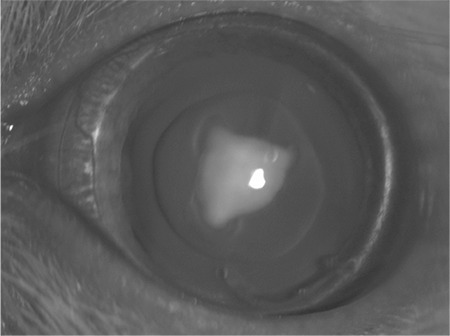
Stage 3 cataract with nuclear lens opacity

**Figure 6:**
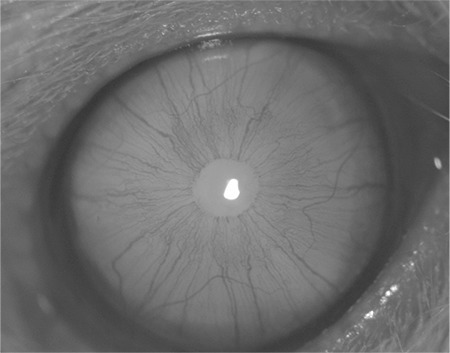
Stage 6 cataract with dense lens opacity

To induce the development of cataract, all newborn rats except those of the control group were administered a single dose (30 nmol/g body weight) of sodium selenite (Sigma Chemical Co., St. Louis, MO, USA) by subcutaneous injection on postnatal day 10. The rats in the control group received no treatment. Between postnatal days 10 and 14, rats in the sham and sesamol groups were intraperitoneally administered 50 mg/kg/day saline solution and 50 mg/kg/day sesamol, respectively. Cataract formation was evaluated by slit-lamp examination at the end of the third week postpartum. Before slit-lamp examination, pupil dilation was induced by instilling 0.5% tropicamide (Tropamid; Bilim, İstanbul, Turkey) and 2.5% phenylephrine hydrochloride drops (Mydfrin; Alcon, Hunenberg, Switzerland) every 30 minutes for 2 hours. All lenses were evaluated and morphologically staged for cataract development (Figures 1, 2, 3, 4, 5, 6). Lens photographs were taken with x25 magnification using a digital camera (CCDIRIS, model DXC 107 AP, Sony Corp., USA) attached to the slit-lamp microscope (Topcon; Tokyo, Japan).

On day 21, after the rats were decapitated under anesthesia, the eyes were enucleated and the lenses were removed with their capsules. Frozen lens samples were weighed and homogenized in ice-cold phosphate buffered saline solution (0.01 M and pH 7.4). Homogenization procedures were carried out using Bullet Blend Tissue Homogenizer (Next Advanced Inc, Averill Park, NY, USA) according to the manufacturer’s instructions at +4 °C. These homogenates were centrifuged at 10,000 x g for 30 minutes at 4 °C, and supernatants were obtained. Supernatants were used for the measurement of the levels of malondialdehyde (MDA), glutathione (GSH), total antioxidant status (TAS), and total oxidant status (TOS).

### Anesthesia Technique

A combination of intramuscular 50 mg/kg ketamine hydrochloride (Ketalar; Eczacıbaşı Holding Co, İstanbul, Turkey) and 6 mg/kg xylazine hydrochloride (Rompun®; Bayer, Turkey) was used for the induction of the anesthesia and analgesia. Before the surgical intervention, a single drop of 1% proparacaine hydrochloride (Alcaine; Alcon Laboratories, Inc., Fort Worth, TX, USA) was instilled in both eyes of each animal.

### Cataract Evaluation and Staging

The development of cataract was evaluated by slit-lamp examination weekly for three weeks. Cataract was graded by the single author (I.E.) according to the slit-lamp appearance of the lenses as described at Hiraoka and Clark12 classification.This classification is as follows:

Stage 0: Normal clear lens,

Stage 1: Initial posterior subcapsular opacity or minimal nuclear opacity,

Stage 2: Slight nuclear opacity with swollen fibers or rare posterior subcapsular opacity,

Stage 3: Cortical radiated diffuse nuclear opacity,

Stage 4: Partial nuclear opacity,

Stage 5: A nuclear opacity not involving lens cortex,

Stage 6: Mature cataract involving the entire lens.

Analysis of MDA, GSH, TAS, and TOS levels in the lenses:

- The level of MDA as an index of lipid peroxidation was analyzed using an MDA kit (Immuchrom GmbH, Hessen, Germany) with high-performance liquid chromatography (HPLC). Initially, protein-bound MDA was hydrolyzed and converted into a fluorescent product (60 min at 95 °C). The fluorescent product was then cooled (2-8 °C), centrifuged, mixed with a reaction solution and injected into the HPLC system. MDA-generated fluorescence was measured in the isocratic HPLC system with a spectrofluorometer detector at 553 nm (emission) and 515 nm (excitation). Results were expressed as nanomoles per milliliter.

- Reduced GSH measurements were carried out using a GSH kit (Immuchrom GmbH, Hessen, Germany) with HPLC. During the derivatization reaction, GSH was converted into a fluorescent probe. The precipitation step removed high molecular weight substances. After centrifugation, the fluorescent probe was cooled (2-8 °C) and a 20 μl sample was injected into the HPLC system. Measurements were carried out on the HPLC system with a fluorescence detector at 385 nm (excitation) and 515 nm (emission). Results were expressed as micromoles per liter.

- TAS and TOS were measured using an automated colorimetric measurement method with commercially available kits (Relassay, Gaziantep, Turkey) on an autoanalyzer (Siemens Advice 2400 Chemistry System, Japan). The principle of the TAS measurement method is based on the oxidation of the 2.2′-azino-bis (3-ethylbenzthiazoline-6-sulphonic acid) (ABTS) molecule to the ABTS+ molecule in the presence of hydrogen peroxide. The rate of the reaction is calibrated with the standard method of Trolox, a vitamin E analog, and its unit is mmol Trolox Equivalent/L.

- The principle of TOS measurement is based on the oxidation of ferrous ion-o-dianisidine complex to ferric ion by the oxidants present in the sample. The color density is correlated with the amount of oxidants in the sample. The spectrophotometric assay method is calibrated with hydrogen peroxide and the results are expressed in terms of micromolar hydrogen peroxide equivalent per liter (µmol H2O2 Equiv/L).

### Statistical Analysis

Statistical analyses were performed using SPSS 22.0 software package (IBM SPSS Statistics software v 22; IBM Corporation, Armonk, NY, USA) to determine the differences between groups. Results are presented as mean ± standard deviation. Normality test was performed for each variable. Analysis of variance (ANOVA) test was used for parametric data fitting a normal distribution. The results were compared between groups with the Mann-Whitney U test, Kruskal-Wallis test, and one-way ANOVA according to the characteristics of the variables. A p value less than 0.05 was considered significant.

## RESULTS

### Cataract Stages

The mean cataract stages of the study groups are shown in Table 1. No cataract development was detected in the control group. The mean cataract stages in the sham and sesamol groups were 3.50±1.41 and 0.57±1.01, respectively. Mean cataract stage in the sham group was significantly higher than in the control group (p<0.05). The mean cataract stage in the sesamol group was significantly lower than that in the sham group (p<0.05).

### MDA, GSH, TAS, and TOS Levels

The mean levels of MDA, GSH, TAS, and TOS in the study groups are shown in Table 1.

The mean MDA levels in the control, sham, and sesamol groups were measured as 4.0±0.46, 12±0.87, and 8±0.51 mmol/L, respectively. Compared to the control group, the sham group had significantly higher levels of MDA, an indicator of lipid peroxidation (p<0.05). The sesamol group had significantly lower MDA levels than the sham group (p<0.05) and significantly higher MDA levels than the control group (p<0.05).

The mean GSH levels obtained from lenses in the control, sham, and sesamol groups were 13±0.90, 6.0±0.15, and 12±0.93 μmol/L, respectively. Mean GSH level was significantly lower in the sham group compared to the control group (p<0.05). The mean GSH level of the sesamol group was significantly higher than that in the sham group (p<0.05). It was observed that the mean GSH level in the sesamol group was not significantly different than that in the control group (p>0.05).

The mean TOS values of the control, sham, and sesamol groups were 121±0.99, 177±0.18, and 148.0±0.22 μmol H_2_O_2_ Equiv./L, respectively. The mean TOS was found to be significantly higher in the sham group compared to the control group (p<0.05). The mean TOS in the sesamol group was significantly lower than that in the sham group (p<0.05). It was observed that the mean TOS in the sesamol group was significantly higher than that in the control group (p<0.05).

The mean levels of TAS in the control, sham, and sesamol groups were 6.75±0.97, 3.09±0.50, and 3.86±0.90 mmol Trolox Equiv./L, respectively. Both the sham and sesamol groups had significantly lower mean TAS levels compared to the control group (p<0.001 and p<0.05, respectively). The mean TAS in the sesamol group was not significantly higher than that in the sham group (p>0.05).

## DISCUSSION

Although various inhibitory or retarder compounds such as vitamins, carotenoids, caffeine, and flavonoids are available, they are not strong enough to completely inhibit cataract formation.^6,13,14^

Various agents such as radiation, galactose, streptozocin, and selenite can be used to experimentally induce cataract formation.^15^ However, we prefer to use selenite for this purpose because cataract induced by selenite is similar in many respects to cataracts found in humans. Selenite was first used by Ostadova et al.^16^ and is currently one of the most commonly used pharmacological agents in experimental cataract models. Selenite causes cataract formation via oxidative damage and lipid peroxidation, generation of hydrogen peroxide, and reduction in the concentration of reduced GSH in the crystalline lens.^15,16^

Sesamol (3.4-methylenedioxyphenol) is the most important antioxidant compound found in sesame oil. In addition to its strong antioxidant activity, sesamol plays a role in a multitude of important biological activities, including induction of growth arrest and apoptosis in cancer cells and cardiovascular cells, enhancement of vascular fibrinolytic capacity, and chemoprevention, and has cytoprotective properties such as radioprotection, gastroprotection, neuroprotection, and myocardial protection; it also exerts antimutagenic, antihepatotoxic, antiplatelet, anti-aging, and anti-inflammatory effects.^17,18,19,20,21,22,23,24,25,26,27,28^

Sesamol potently and significantly decreases hydroxyl radical generation and lipid peroxidation. Sesamol has been found to reduce the activity of monoamine oxidase (MAO) and the generation of nitrite oxide and hydrogen peroxide in glial astrocytes.^11,17,18,19,20,21,22,23,24,25,26,27,28^ Therefore, it has neuroprotective effects against cerebral ischemia.^29^ MAO is an enzyme that catabolizes certain amines in the brain, such as serotonin, dopamine, and norepinephrine. Thus, it has been suggested that sesamol might play a protective role in age-related neurodegenerative disorders such as Alzheimer’s disease and stroke.^30^ A recent study has reported that sesamol was found to have a protective effect against experimental diabetic nephropathy via reduction of lipid peroxidation.^21^

GSH, or L-γ-glutamyl-L-cysteinyl-glycine, which is synthesized by the lens epithelium, plays an extremely important role in protecting the lens from oxidative damage. The intracellular GSH level is regulated by an enzyme complex compound consisting of GSH synthase, GSH peroxidase, and GSH reductase.31,32 Additionally, nicotinamide adenine dinucleotide phosphate-oxidase and glucose-6-phosphate dehydrogenase, which are the main functional enzymes in GSH synthesis, slow senile cataract formation via sweeping oxidant action.33 Many studies have shown that GSH levels are high in cataractous lenses, but low in normal lenses. Thus, elevated intracellular GSH level leads to lipid peroxidation and damage of multiple cellular systems by free radicals.^32,33^

MDA is the main metabolite generated by lipid oxidation in the cells, and it might change the function and activity of DNA and proteins by crosslinking them. Membrane phospholipids and low-density lipoprotein are the macromolecules that are most susceptible to the effects of free radicals.^34^ Oxidation of the double bonds in unsaturated fatty acids due to lipid peroxidation causes deterioration in membrane permeability, membrane fluidity, and the swing function in membrane disorders.^35^

Plasma contains many antioxidant compounds such as bilirubin, free iron-bearing transferrin, ceruloplasmin, uric acid, vitamin E, vitamin C, and proteins, and distributing these throughout the body is a critical function of the blood.^36^ TAS is the most important factor in plasma. The antioxidants in plasma are constantly interacting and potentiating each other’s effects; in addition, a decrease in one type of antioxidant can be compensated by an increase in others. The measurement of TAS provides more valuable information than can be obtained from measurements of single antioxidants. Therefore, total antioxidant capacity is measured to determine antioxidant status instead of the values of single antioxidant levels.^35,36^

## CONCLUSION

To the best of our knowledge based on our literature search of the PubMed database, no previous study has examined the use of sesamol in the prevention of cataract development in any experimental cataract model. Thus, our report is the first to address this subject. The low TOS and MDA values and high TAS and GSH values obtained in our study suggest that the antioxidant effects of sesamol might inhibit cataract formation. Further research is needed to determine the potential antioxidant effects of these agents in humans.

## Figures and Tables

**Table 1:**
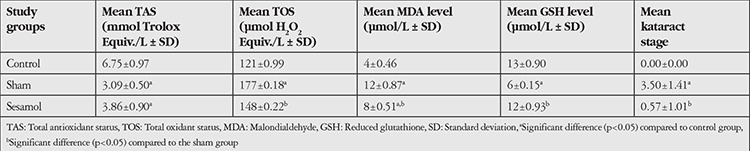
The levels of total antioxidant status, total oxidant status, malondialdehyde, glutathione and the stages of cataract development in the study groups
